# Intra-colony disease progression induces fragmentation of coral fluorescent pigments

**DOI:** 10.1038/s41598-017-15084-3

**Published:** 2017-11-03

**Authors:** Jamie M. Caldwell, Blake Ushijima, Courtney S. Couch, Ruth D. Gates

**Affiliations:** 1 Hawai‘i Institute of Marine Biology, School of Ocean and Earth Science and Technology, University of Hawai‘i at Mānoa, Kāne‘ohe, HI 96744 United States; 2 Department of Microbiology, University of Hawai‘i at Mānoa, Honolulu, HI 96822 United States; 3 Present Address: Department of Biology, Stanford University, Stanford, CA 94305 United States; 4 Present Address: Department of Biomedical Sciences, College of Veterinary Medicine, Oregon State University, Corvallis, OR 97331 United States

**Keywords:** Wide-field fluorescence microscopy, Infection, Animal physiology

## Abstract

As disease spreads through living coral, it can induce changes in the distribution of coral’s naturally fluorescent pigments, making fluorescence a potentially powerful non-invasive intrinsic marker of coral disease. Here, we show the usefulness of live-imaging laser scanning confocal microscopy to investigate coral health state. We demonstrate that the Hawaiian coral *Montipora capitata* consistently emits cyan and red fluorescence across a depth gradient in reef habitats, but the micro-scale spatial distribution of those pigments differ between healthy coral and coral affected by a tissue loss disease. Naturally diseased and laboratory infected coral systematically exhibited fragmented fluorescent pigments adjacent to the disease front as indicated by several measures of landscape structure (e.g., number of patches) relative to healthy coral. Histology results supported these findings. Pigment fragmentation indicates a disruption in coral tissue that likely impedes translocation of energy within a colony. The area of fragmented fluorescent pigments in diseased coral extended 3.03 mm ± 1.80 mm adjacent to the disease front, indicating pathogenesis was highly localized rather than systemic. Our study demonstrates that coral fluorescence can be used as a proxy for coral health state, and, such patterns may help refine hypotheses about modes of pathogenesis.

## Introduction

Natural fluorescence is ubiquitous in scleractinian corals, although their biological function remains unresolved. Coral pigmentation is produced by fluorescent proteins found in coral tissue (i.e., green fluorescent protein and its homologues)^1–3^ and photosynthetic pigments produced by symbiotic dinoflagellates, *Symbiodinium* spp. (i.e., chlorophyll-*a*, chlorophyll c_2_, peridinin, diadinoxanthin, diatoxanthin, β,β-carotene, dinoxanthin)^4^. The functional role(s) of fluorescent proteins (FPs) in coral is currently unclear; however, there are several leading hypotheses. The most well supported hypothesis suggests that coral FPs are photoprotective in shallow-water habitats, dissipating energy in excessive sunlight^5,6^, and photo-enhancing in deep-water habitats, increasing light absorption in low light conditions^7^. Other studies suggest FPs may also play a role in immune function, including response to stressful temperatures^8^, mechanical damage, infestation, infection, and tissue regeneration^9–12^.

Despite a limited understanding of the function of fluorescence, natural fluorescence has been used as a non-invasive intrinsic marker of coral physiological condition and may be particularly suitable for investigating coral disease. In laboratory experiments, coral fluorescence has been used as a proxy for photochemical efficiency and growth in response to temperature stress, wounding, epibiont infestation, and recovery^9–11^. There is evidence that fluorescence may be involved in other facets of the innate immune system such as inflammatory responses typical of coral diseases^9,11^ or tissue regeneration^12^, which is important for recovery from infection. However, no studies have investigated a fluorescence response to pathogen infection in a controlled laboratory experiment. In this study, we characterize changes in fluorescence associated with a coral disease response using field collected diseased fragments and a laboratory inoculation experiment.

We explored the usefulness of a new technology, live-imaging laser scanning confocal microscopy, as a tool for characterizing coral health state based on the signature of endogenous fluorescence. Previous studies have used total fluorescence concentration or expression of fluorescent genes as a proxy for coral physiological condition (e.g., refs^6,12,13^), which can be detected before physiological changes can be seen with the naked eye. Monitoring sub-lethal stress is particularly important for diseases because once a visual lesion manifests the coral is already experiencing cell death. Confocal microscopy provides an opportunity to explore multiple facets of fluorescence in living coral, including the identification of different colors being simultaneously expressed and the spatial distribution of those pigments across fine spatial scales. Disease-induced changes in the colors and/or spatial distributions of fluorescent pigments could support different hypotheses about the coral immune response, modes of pathogen infection, and intra-colony disease progression. For instance, different patterns of fluorescence may arise from diseases that move across a colony surface versus diseases that move through the gastrovascular canal versus diseases that directly attack coral symbionts.

As a case study, we quantified disease-induced changes in coral fluorescence associated with tissue loss diseases in *Montipora capitata*, a common reef-building coral found throughout Hawai‘i. *M. capitata* is affected by both a slow-moving tissue loss disease referred to as chronic *Montipora* white syndrome, which is found year-round, and a rapid tissue loss disease referred to as acute *Montipora* white syndrome, which results in short-term outbreak events often in winter months^14,15^. Both forms of this disease manifest as tissue degradation with a clear boundary between apparently healthy tissue and exposed skeleton. In Hawai‘i, *Vibrio owensii* and *Vibrio coralliilyticus* has been shown to cause chronic and acute *Montipora* white syndrome respectively^16,17^. The mode of pathogen infection it is currently unclear, and, secondary invaders such as ciliates, helminthes, and chimeric parasites, which cause different types of injury in the coral host, could support one of two contradictory modes of intra-colony disease spread: localized or systemic^18^. If disease spread is localized to the coral surface, we hypothesized there would be fragmentation of fluorescence near the point of pathogen entry, indicating a loss in tissue structure and ability to translocate nutrients in the affected area. If disease spread is systemic, we hypothesized that changes in fluorescence would be restricted to coral polyps, potentially indicating that the pathogen is consumed heterotrophically and moves freely through the gastrovascular canal. Systemic spread would support an *in vivo* study by Shapiro *et al*.^19^, which found concentration of *Vibrio coralliilyticus* around coral polyps and gastroderm^20^.

To determine if fluorescence is a viable physiological proxy for coral health and intra-colony disease spread, we conducted four experiments to investigate spatially explicit patterns of natural fluorescence in healthy and diseased *M. capitata*. Building on previous research efforts exploring the wide range of fluorescence signatures found within and between coral species in different habitats and environmental conditions^21–23^, we first characterized the natural variability in *M. capitata* fluorescence across a depth gradient. We then compared this emission signature (types and distribution of pigments) to the fluorescent patterns from naturally diseased and laboratory inoculated coral fragments. We complemented this analysis with histology to determine whether differences in fluorescence were attributed to patterns in the distribution of *Symbiodinium*.

## Results

### Natural variability in coral fluorescence across habitats

**Figure 1:**
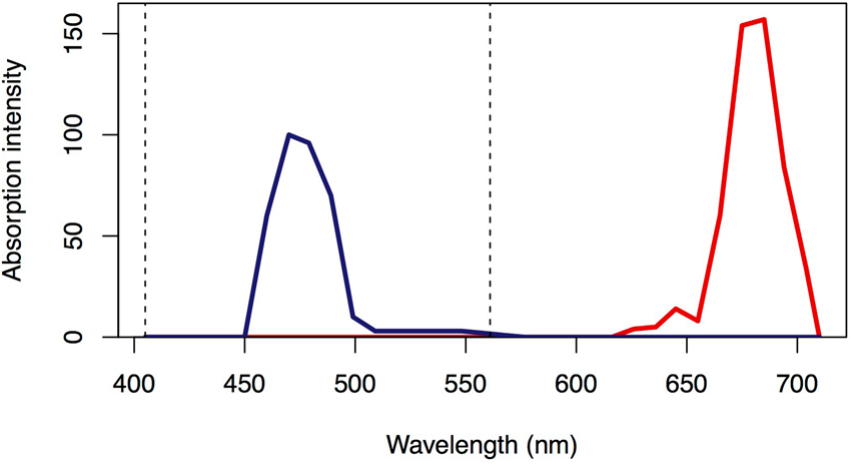
Emission spectra for *Montipora capitata*. Cyan fluorescence (blue solid line) and red fluorescence (red solid line) emission spectra. Vertical dotted lines indicate excitation spectra.

**Figure 2:**
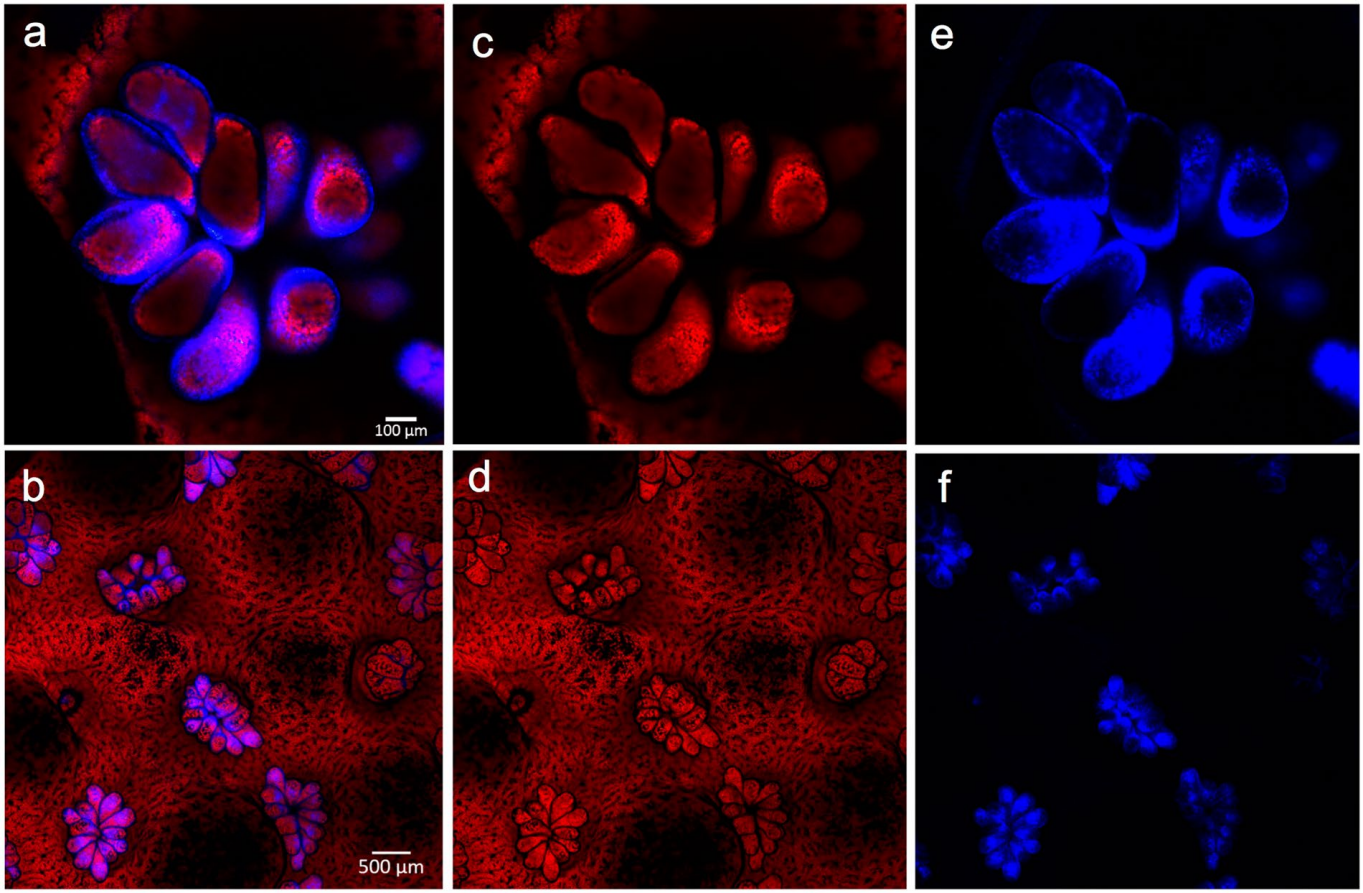
Confocal microscopy at two spatial scales. Confocal images of living healthy *M. capitata* fragments at the (**a**) polyp scale (10x objective) and (**b**) branch scale (25x objective). Each image was separated into two layers corresponding to dominant emission spectra: red (middle panel), and cyan (right panel).

Spectral signatures were similar for visually healthy coral fragments from all three depths (1 m, 3 m and 5 m). Coral fragments emitted cyan fluorescence between 460 and 495 nm and red fluorescence between 650 and 680 nm in response to simultaneous excitation at 405 nm and 561 nm (Fig. 1). These emission spectra resemble the emission spectra of cyan fluorescent protein (483 nm – 495 nm found in^3,5,23^) and chlorophyll-*a* (peak ~685 nm found in ref.^24^). Cyan fluorescence was primarily restricted to the coral polyps whereas red fluorescence was distributed across the coral polyps and coenosarc (tissue overlying the coral skeleton; Fig. 2a,b). When separating emission spectra (Fig. 2c–f), we found no significant difference between the ratio of cyan to red fluorescence at the polyp scale or at the branch scale based on results from one-way analysis of variance (ANOVAs) with depth as a factor. The spatial distribution of fluorescent pigments revealed distinctly different patterns between coenosarc and polyps (Fig. 2a,b), and those patterns were consistent across all fragments within and between depths. We found no significant difference in the number of intact polyps across depths at the branch scale based on results of a one-way ANOVA. We also found no differences in the spatial distribution of fluorescent pigments across depths at the branch scale in any of the five metrics of landscape structure we evaluated using one-way ANOVAs: total area of fluorescence, edge area, edge to area ratio, number of patches (contiguous breaks in fluorescence), and area of patches.

### Differences in fluorescence between healthy and naturally diseased coral

**Figure 3:**
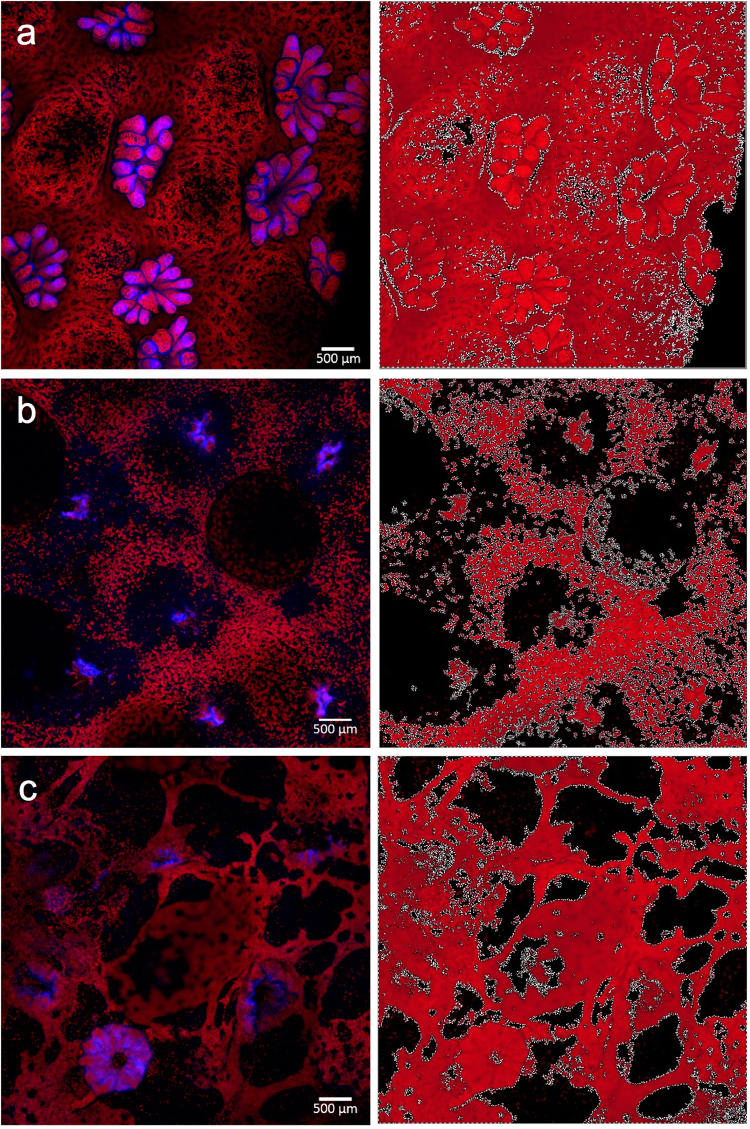
Image analyses to characterize landscape structure. Confocal microscopy images of (**a**) healthy, (**b**) field collected diseased, and (**c**) laboratory inoculated coral fragments, which were analyzed in Photometrica 7.0 to characterize five measures of landscape structure: total area of fluorescence, edge area, edge to area ratio, number of patches, and patch area. Confocal microscopy images are shown in images on the left, Photometrica analyses of the microscopy images are shown in images on the right. In Photometrica images, the area highlighted in red reflects total area of fluorescence; black regions indicate patches, and white lines indicate edges.

**Figure 4:**
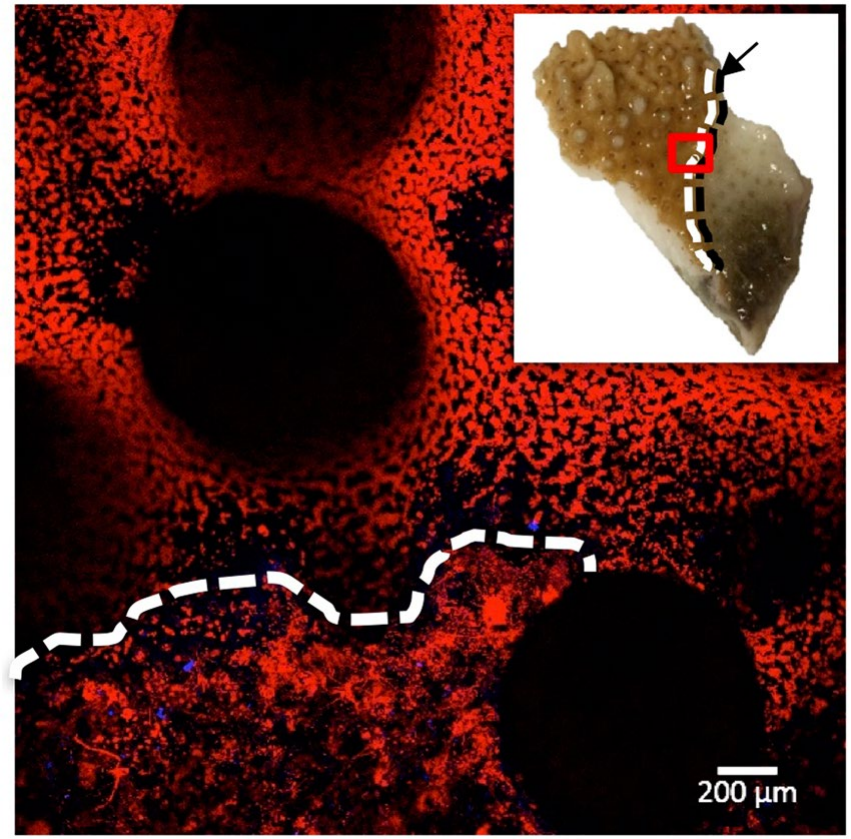
Linear extension of disease front. Microscopy image of diseased coral fragment. White dotted line indicates the upper limit of fragmented fluorescence adjacent to the disease front. Inset image shows the diseased coral fragment, with a red box indicating the microscope image area, a black arrow and dotted line indicating the visual disease front, and a white dotted line corresponding to the microscopy image of the upper limit of fragmented fluorescence.

While there was no differences in the emission spectra (fluorescent colors) of visually healthy and diseased coral fragments, the spatial pattern of fluorescent pigments differed by most landscape metrics that we tested based on t-tests and Wilcoxon rank-sum tests (differences illustrated in Fig. 3a versus 3b), including total area of fluorescence, edge area, edge to area ratio, and number of patches (see Methods for details). These metrics of landscape structure were used as proxies for fragmentation, which indicates tissue coverage and connectedness. Fragmentation of the connective coral tissue would impede the coral’s ability to prevent pathogen entry and the translocation of critical coral nutrients. In diseased fragments, the average area of affected fluorescence extended 3.03 mm ± 1.80 mm beyond the disease front (visual lesion edge; Fig. 4). The ratio of cyan to red fluorescence refers to the comparison between the area of cyan fluorescence and red fluorescence in an image and did not differ at the polyp or branch scale in healthy and diseased fragments (Table 1). In contrast, the spatial distribution of fluorescent pigments at the branch scale indicated significant differences between healthy and diseased fragments (Table 1; Fig. 3a,b). Mean area of fluorescence was 1.2 times greater in healthy fragments compared to diseased fragments (t = −2.7, df = 29, p = 0.01; Table 1) and there were 3.4 times as many intact polyps in healthy fragments compared to diseased fragments (t = −4.9, df = 26, p = 0.002; Fig. 3a,b). On average, diseased fragments had 1.4 times greater edge area (t = 2.8, df = 30, p = 0.01; Tables 1), 1.8 times greater edge to area ratio (t = 3.6, df = 22, p = 0.002; Table 1) and 1.5 times as many patches relative to healthy fragments (t = 2.5, df = 32, p = 0.02; Table 1). There was no significant difference between patch area in healthy and diseased fragments.

**Table 1:** Analysis of spatial distribution of fluorescent pigments and ratio of fluorescence emission spectra.

Variable	Healthy group mean	Diseased group mean	Degrees of freedom	P-value
Total area fluorescence (mm)	44.00	37.00	29	<0.01
Number of intact polyps	5.40	1.60	6	<0.01
Edge area (mm)	5.80	8.00	30	<0.01
Number of patches	3.17	4.82	32	<0.05
Area of patches* (mm)	0.02	0.05	—	>0.05
Edge:area ratio	0.14	0.25	22	<0.01
Cyan:red fluorescence ratio*	0.00	0.05	—	>0.05
Cyan:red fluorescence ratio*^†^	44.00	3.90	—	>0.05

*Non-parametric Wilcoxon rank sum test. ^†^Measurement taken using 10x objective (polyp scale).

### Histological investigation of visually healthy and diseased coral

Mean *Symbiodinium* density in visually healthy fragments (22.71 ± 3.08 per contour length of gastrodermis within the surface and basal body walls) did not differ from diseased fragments (17.57 ± 3.26) (t = 0.96, df = 20, p = 0.35). However, density was higher in the surface (27.71 ± 3.26) compared to the basal (14.04 ± 2.07) body wall (t = 3.6, df = 26, p = 0.001). Symbiont abundance per contour length of surface body wall did not change with distance from the lesion margin in naturally diseased corals (*R*^2^ = −0.022, p = 0.92).

### Differences in fluorescence between healthy and laboratory infected coral

**Figure 5:**
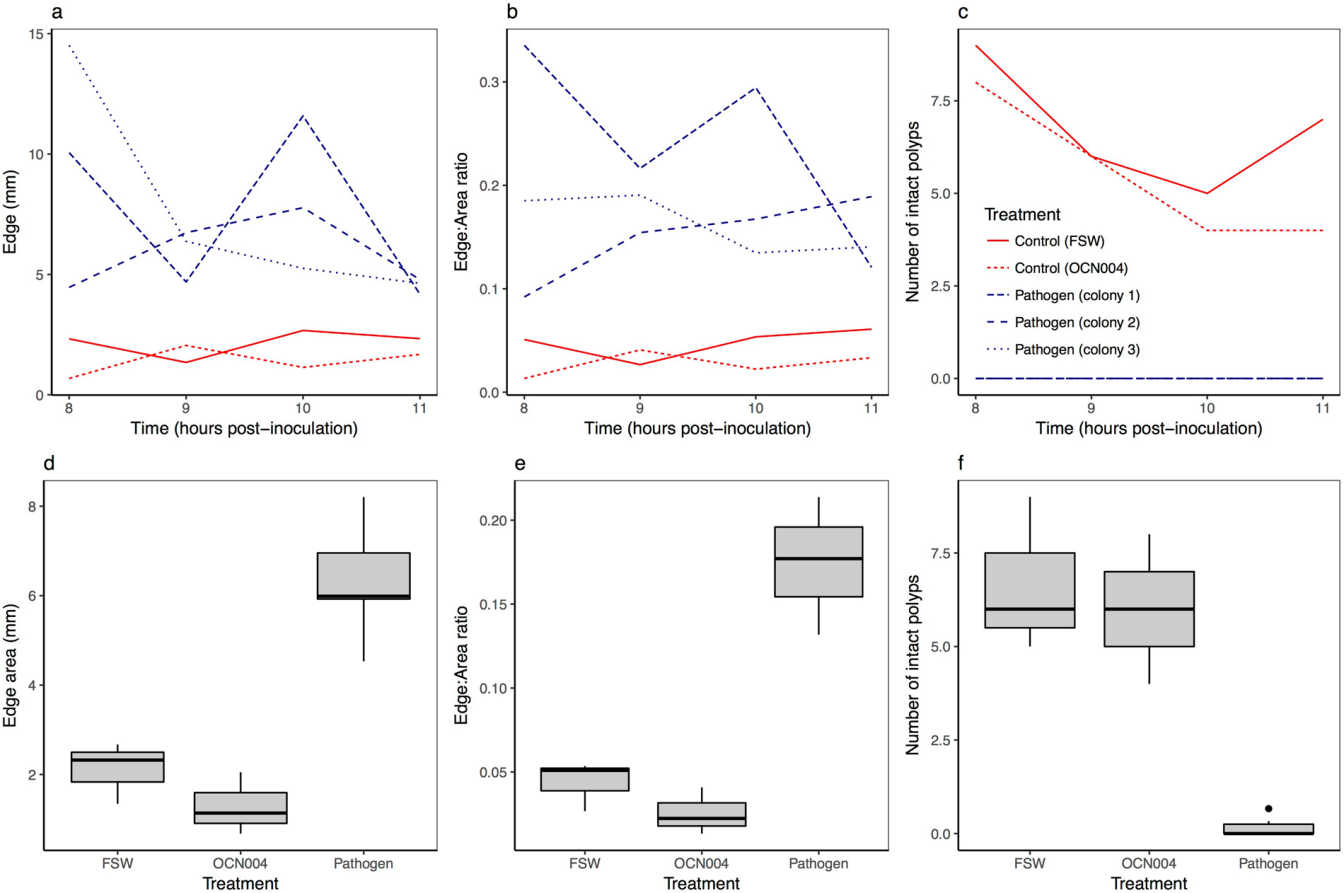
Differences in landscape structure between healthy controls and laboratory inoculated corals. Line graphs (**a**–**c**) show high intra-specific variability within treatments where FSW represents fresh seawater and OCN004 represent the negative bacterial control, for (**a**) mean edge area, (**b**) mean edge to area ratio, and (**c**) mean number of intact polyps within microscopy image (~1 cm^2^). Boxplots (**d**–**f**) show significant differences across treatments in (**d**) mean edge area, (**e**) mean edge to area ratio, and (**f**) number of intact polyps within microscopy image (~1 cm^2^).

We found significant differences in fluorescent pigment distribution between healthy and laboratory inoculated coral. Inoculated coral fragments showed similar patterns of fluorescence to those of naturally diseased coral fragments (Fig. 3b,c); however, there was high individual variability in the rate of disease progression, obscuring any distinct temporal changes in fluorescence associated with intra-colony disease spread (Fig. 5a–c). We found no significant differences in emission spectra or the spatial distribution of fluorescent pigments between fragments sampled between 8 and 11 hours post-inoculation using repeated measures mixed effects models. On average, visual lesions developed in hour 10 post-inoculation. Combining inoculated fragments from all time points, we compared spectral and landscape metrics between fresh seawater control, negative bacterial control, and pathogen treatments based on two-way ANOVAs with treatment and colony as random effects. The ratio of cyan to red fluorescence did not differ at the polyp or branch scale, and there were no differences across treatments in total area of fluorescence, number of patches, or patch area. Mean edge area (F_df=2,df=5_ = 39.4, p = 0.002) and edge to area ratio (F_df=2,df=5_ = 109.7, p = 0.0003) were higher and number of intact polyps (F_df=2,df=5_ = 39.7, p = 0.002) was lower in the inoculated treatment compared to control groups (Fig. 5d–f).

## Discussion

Our research provides proof-of-concept for the utility of live-imaging laser scanning confocal microscopy for investigating coral disease, laying the foundation to investigate disease for other coral species and different types of diseases.

Confocal microscopy images of *Montipora capitata* fluorescence patterns were consistent for visually healthy coral across a range of depths, but systematically differed between healthy and diseased coral. Similar to other species^24,25^, our results suggest that healthy *M. capitata* has a unique composition of endogenous fluorescence (type and arrangement of fluorescent pigments), and that composition is maintained across shallow water habitats. Differences in the spatial distribution of fluorescent pigments between healthy and diseased coral indicated sub-lethal changes in coral tissue that cannot be seen in natural light conditions with the human eye. Our results show that healthy coral fluorescence patterns were structured and predictable, whereas diseased coral fluorescence was disorganized and fragmented within a boundary directly adjacent to the visual disease lesion (Fig. 4). In this disease front boundary, there was less total coverage of fluorescence and the fluorescent pigments were not distributed equally across the coral, suggesting a disruption in the coenosarc and gastrodermis, which may impede translocation of energy between polyps. Beyond the disease front boundary, polyps had distended or fractured tentacles, or were completely disintegrated (indicated by empty corallites), which inhibits heterotrophic feeding. The sub-lethal coral disease response extended directly from the disease front suggesting that pathogenesis is highly localized, potentially spreading across the tissue surface, which can be tested in future studies. This study builds on other research efforts that show fluorescence expression is altered in response to temperature stress, wounding, epibiont infestation, and recovery^10,12,13^, allowing for the use of fluorescence as a proxy for coral health state.

Histology showed intact, apparently healthy *Symbiodinium* in the gastrodermis supporting the hypothesis that the *Symbiodinium* maintain their physiological function while the coral is infected, which supports similar findings for *Acropora* white syndrome on the Great Barrier Reef^26^. However, Roff *et al*.^26^ found that the abundance of *Symbiodinium* in *Acropora* white syndrome was maintained in the coral tissue directly adjacent to the disease lesion. In contrast, we found that the total area of red fluorescence decreased directly adjacent to and several millimeters away from the disease front in chronic and acute *Montipora* white syndrome. Histological analyses revealed that *Symbiodinium* density per unit area of gastrodermis was not reduced with distance from the lesion, thus, the decrease in red fluorescence reflects less coverage of the gastrodermis overall rather than a loss of symbionts. We also found that symbiont density was significantly higher in the surface body wall compared to the basal body wall suggesting that *Symbiodinium* are not migrating deeper into the tissue.

While we were able to reproduce the fluorescence response in laboratory inoculations, we found large individual variation in the rate of disease progression, making it difficult to investigate disease patterns through time (Fig. 5a–c). The high variability across time points could have been associated with a low sample size, use of replicate fragments, and/or intraspecific variability from wild coral populations. Despite high variability among fragments, field and inoculation experiments demonstrated the same overall trend where diseased fragments elicited pigment fragmentation. Furthermore, comparison between the two experiments led to interesting results: 1) pathogen inoculated coral had moderate edge area compared with healthy and naturally diseased fragments, and 2) negative control fragments (OCN004) had low edge area compared to healthy field collected fragments. There are two explanations that could support the differing results between edge area in pathogen inoculated coral versus healthy and diseased field collected coral, where the differences reflect: i) distinctions between chronic *Montipora* white syndrome (naturally diseased coral) and acute *Montipora* white syndrome (laboratory inoculated coral) that can be caused by different pathogens^16,17,27^; or 2) differences in the timing of microscopy, as we imaged laboratory inoculated corals within one day of infection, whereas we did not know how long field collected colonies had been infected at the time of imaging. In contrast, we hypothesize the low edge area in negative control fragments was due to a low sample size, given that the low edge area was not outside the range of edge area for healthy field-collected fragments.

Our study lays the foundation to address several other questions regarding disease progression using natural fluorescence as a proxy of health state for coral or for other naturally fluorescent organisms. In this study, we were interested in how coral responds to tissue loss diseases. Our results indicate that healthy and diseased coral have distinct patterns of fluorescent pigment distributions. This approach could be used for other coral diseases that illicit a fluorescence response. Future studies could explore temporal trends in infection, investigate mode of infection using fluorescently tagged pathogenic bacteria, and compare fluorescence responses across a variety of coral diseases and species, or even investigate disease responses in other naturally fluorescent organisms. Investigating changes in the spatial arrangement of fluorescent pigments is a new approach to assess disease lesions and within-individual stress responses. Our results indicate that confocal microscopy is a powerful tool that can complement more commonly used disease methods such as histology. A key advantage for this technique is that confocal microscopy allows researchers to study *living* organisms, and thus, it can be used to study physiological changes within individuals over time and between individuals across different treatments.

## Methods

### Collection of coral fragments

For all experiments in this study, we collected coral fragments from red, branching *Montipora capitata* colonies between 25 and 35 cm maximum length from Coconut Island in Kāne‘ohe Bay, O‘ahu, Hawai‘i. All fragments were approximately 4 cm^2^ and placed in a water table for acclimation at least 24 hours prior to microscopy. To capture the natural variability in healthy *M. capitata* coral fluorescence, we collected five replicate fragments from one, three and five meter depths, for a total of 15 colonies. To characterize and compare fluorescence between healthy and naturally diseased colonies, we collected fragments at ~2 m depth from 16 colonies exhibiting signs of chronic *Montipora* white syndrome. Each diseased fragment was taken from the disease front, and therefore included partially healthy and partially diseased tissue. To compare coral fluorescence with histology, we collected six pairs of healthy and diseased coral fragments (colonies located within 1 m of each other) at ~2 m depth. Paired fragments were scarified for histological investigation after confocal microscopy. For laboratory inoculations, we collected seven replicate fragments from three visually healthy *M. capitata* colonies for a total of 21 fragments.

### Histology

After we collected visually healthy and diseased *M. capitata* coral fragments from the field, they were placed in Whirl-paks®, imaged under the confocal microscope and then immediately fixed in 1:4 zinc-buffered formalin (Z-Fix Concentrate, Anatech, Ltd.) diluted with artificial seawater for histological analysis. All fixed samples were rinsed thoroughly in deionized water and then decalcified in 1% formic acid for 1 to 2 days followed by 2% formic acid for another 1 to 2 d or until fully decalcified. Samples were trimmed to 3 cm^2^, placed in cassettes, rinsed thoroughly and then placed in 70% ethanol. Samples were embedded in paraffin wax, cross sectioned at 5 μm thickness and mounted on slides at Histo Techniques, LTD. De-paraffinized sections were stained with hematoxylin and eosin and cover-slipped prior to light microscopy. To determine whether *Symbiodinium* density varied between health states or location within the tissue, *Symbiodinium* were counted along the contour length of gastrodermis within the surface and basal body walls for all coral fragments. For diseased tissue, *Symbiodinium* were quantified in the intact tissue directly adjacent to the lesion margin. We normalized counts of zooxanthellae by dividing numbers of each of *Symbiodinium* by contour length (i.e., number of *Symbiodinium* per unit length of tissue) for each health state and location within the tissue (surface or basal body wall).

### Laboratory inoculations

To compare healthy coral with disease lesions in naturally diseased and laboratory infected coral, and, to monitor changes in coral fluorescence over time during an infection, we conducted laboratory inoculations. Experimental set up and bacterial inoculation was conducted as previously described^16^. Briefly, for each of the three replicate colonies (with seven replicate fragments per colony), four fragments were inoculated with a bacterial pathogen (*Vibrio coralliilyticus* strain OCN008), one fragment was inoculated with the non-pathogenic negative control bacterium *Alteromonas* sp. strain OCN004, one fragment was kept in an aquarium with only filtered seawater, and one fragment was left untreated after sampling and kept in a flow-through water table. Coral fragments were inoculated in aquaria and remained in the tanks with pathogenic bacteria over the course of the experiment. Complementary experiments indicate that OCN008 concentration stays consistent over time in tank experiments with and without coral, while OCN008 concentration on coral tissue increases until visible lesions appear (Ushijima and Häse, unpublished data). OCN008 is a strain of *Vibrio coralliilyticus* known to cause acute *Montipora* white syndrome^17^. To limit the handling time of any one coral fragment, we imaged a unique replicate fragment inoculated with the bacterial pathogen at 8, 9, 10, and 11 hours post-inoculation (in contrast to repeatedly imaging the same coral fragment at each sampling time point). Aquaria were maintained at 25 °C under ambient sunlight. The final inoculum concentration used, for both the pathogen and control bacterium, was calculated to be 10^8^ CFU per ml of aquarium water.

All marine bacteria were grown in a modified version of glycerol artificial seawater (GASW) media^17^, which was supplemented with 15 g/l of agar prior to autoclaving for solid media. Marine bacteria were kept in cryopreservation at −80 °C until needed, at which point stocks were streaked out onto GASW plates and incubated overnight at 29 °C. Colonies from this plate were used to start liquid culture inoculation of coral fragments.

### Confocal microscopy

We imaged natural fluorescence of living coral fragments using a Zeiss LSM 710 live-imaging laser scanning confocal microscope (LSCM). We used excitation lasers at 405 nm and 561 nm and captured emission spectra at 32 wavelengths between 405 and 755 nm. To characterize overall diversity of natural fluorescence and to spatially localize the arrangement of fluorescent pigments, we imaged coral samples at two spatial scales corresponding to the coral branch (image coverage of ~50 mm^2^ using a 2.5x objective) and the coral polyp (image coverage of ~1 mm^2^ using a 10x objective).

### Image analysis

We quantified the relative area and spatial distribution of fluorescent pigments in confocal microscopy spectral images using Photometrica 7.0 (Westboro Photonics). We parsed each confocal image into separate layers corresponding to fluorescence produced in the red and cyan emission spectrum (Fig. 2c–f), and, within each layer, delineated and classified key physiological features using Area of Interest (AOI) classifiers. To quantify the relative contribution of each dominant spectra, we calculated the ratio between the total area of cyan and red fluorescence within specified AOIs (i.e., polyp, branch). To quantify the spatial distribution of fluorescent pigments in each image at the branch scale (25x objective), we measured five metrics of landscape structure: total area of fluorescence (number of contiguous pixels greater than 400 pixels with luminosity greater than 0.22 Watts), edge area (1-pixel-wide area surrounding contiguous patches of fluorescence), edge to area ratio, number of patches (number of patch AOIs within a branch AOI; corresponds to black areas in the right panel of Fig. 3), and patch area. At the branch scale, we also counted the number of intact polyps based on tentacle and oral disk structure.

### Statistical analyses

We analyzed the ratio of cyan to red fluorescence and metrics of landscape structure from the image analysis in R statistical software v 3.3.1. We compared fluorescence across habitats using one-way analysis of variance tests (ANOVAs) and across inoculation treatments and colony using two-way ANOVAs. We pooled healthy samples from all depths and compared visually healthy and naturally diseased coral using two-sample t-tests for normally distributed and/or transformed variables with normal distributions and Wilcoxon rank sum tests for non-normally distributed variables (Table 1). To compare *Symbiodinium* density between visually healthy and naturally diseased corals and between the surface and basal body walls, we first log transformed *Symbiodinium* density and then conducted paired t-tests. To compare fluorescence over time for the inoculation experiment, we conducted repeated measures mixed effects models on replicate fragments from the same coral colony imaged over time.

### Data availability

The data generated during and/or analyzed in the current study are available from the corresponding author on reasonable request.
