# Publisher Correction: Intra-colony disease progression induces fragmentation of coral fluorescent pigments

**DOI:** 10.1038/s41598-017-17987-7

**Published:** 2018-01-05

**Authors:** Jamie M. Caldwell, Blake Ushijima, Courtney S. Couch, Ruth D. Gates

**Affiliations:** 1 Hawai‘i Institute of Marine Biology, School of Ocean and Earth Science and Technology, University of Hawai‘i at Mānoa, Kāne‘ohe, HI 96744 United States; 2 Department of Microbiology, University of Hawai‘i at Mānoa, Honolulu, HI 96822 United States; 3 Present Address: Department of Biology, Stanford University, Stanford, CA 94305 United States; 4 Present Address: Department of Biomedical Sciences, College of Veterinary Medicine, Oregon State University, Corvallis, OR 97331 United States

Correction to: *Scientific Reports* 10.1038/s41598-017-15084-3, published online 03 November 2017

The original HTML version of this Article contained a typographical error in the publication date ‘03 November 2017’ which was incorrectly given as ‘06 November 2017’. This has now been corrected in the HTML version of the Article.

