# Publisher Correction: Intra-colony disease progression induces fragmentation of coral fluorescent pigments

**DOI:** 10.1038/s41598-018-22375-w

**Published:** 2018-03-06

**Authors:** Jamie M. Caldwell, Blake Ushijima, Courtney S. Couch, Ruth D. Gates

**Affiliations:** 1 Hawai‘i Institute of Marine Biology, School of Ocean and Earth Science and Technology, University of Hawai‘i at Mānoa, Kāne‘ohe, HI 96744 United States; 2 Department of Microbiology, University of Hawai‘i at Mānoa, Honolulu, HI 96822 United States; 3 Present Address: Department of Biology, Stanford University, Stanford, CA 94305 United States; 4 Present Address: Department of Biomedical Sciences, College of Veterinary Medicine, Oregon State University, Corvallis, OR 97331 United States

Correction to: *Scientific Reports* 10.1038/s41598-017-15084-3, published online 03 November 2017

An error occurred after publication resulting in the inadvertent omission of the Article and Volume numbers from the PDF version of this Article. This error has now been corrected in the PDF version of the Article; the HTML version was correct from the time of publication.

